# Intrinsic factors driving mosquito vector competence and viral evolution: a review

**DOI:** 10.3389/fcimb.2023.1330600

**Published:** 2023-12-21

**Authors:** Juliette Lewis, Emily N. Gallichotte, Jenna Randall, Arielle Glass, Brian D. Foy, Gregory D. Ebel, Rebekah C. Kading

**Affiliations:** ^1^ Center for Vector-borne Infectious Diseases, Department of Microbiology, Immunology, and Pathology, Colorado State University, Fort Collins, CO, United States; ^2^ Department of Cellular and Molecular Biology, Colorado State University, Fort Collins, CO, United States

**Keywords:** transmission, barriers, arbovirus, antiviral immunity, microbiome, genomics, applications

## Abstract

Mosquitoes are responsible for the transmission of numerous viruses of global health significance. The term “vector competence” describes the intrinsic ability of an arthropod vector to transmit an infectious agent. Prior to transmission, the mosquito itself presents a complex and hostile environment through which a virus must transit to ensure propagation and transmission to the next host. Viruses imbibed in an infectious blood meal must pass in and out of the mosquito midgut, traffic through the body cavity or hemocoel, invade the salivary glands, and be expelled with the saliva when the vector takes a subsequent blood meal. Viruses encounter physical, cellular, microbial, and immunological barriers, which are influenced by the genetic background of the mosquito vector as well as environmental conditions. Collectively, these factors place significant selective pressure on the virus that impact its evolution and transmission. Here, we provide an overview of the current state of the field in understanding the mosquito-specific factors that underpin vector competence and how each of these mechanisms may influence virus evolution.

## 1 Introduction

**Figure 1:**
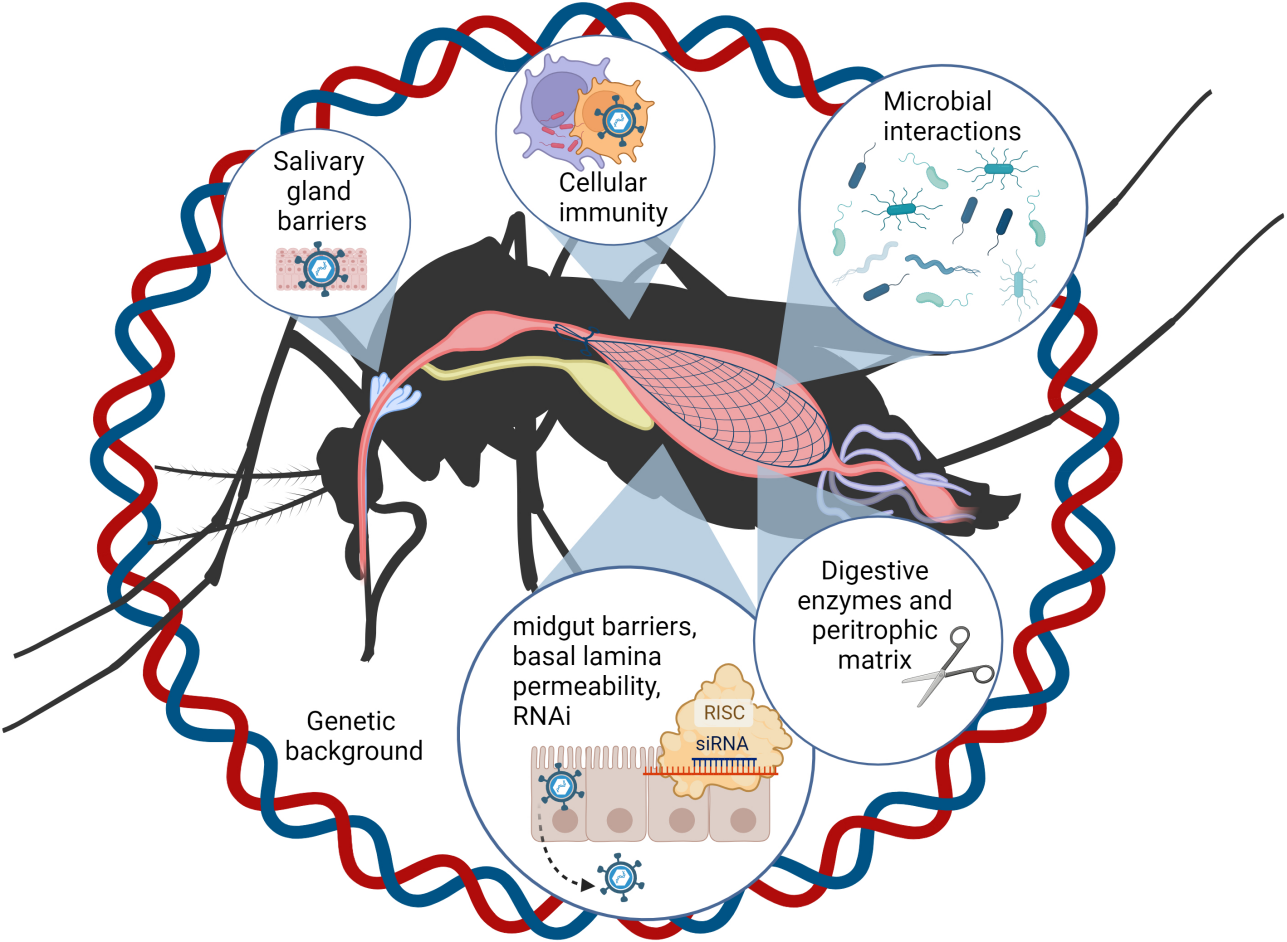
Numerous factors inherent to the mosquito influence vector competence and viral evolution. For successful horizonal transmission (by bite), ingested viral particles must enter and replicate in the midgut epithelial cells, exit the midgut and pass through the body cavity or hemocoel to invade the salivary glands, and be expelled with the saliva when the vector takes a subsequent blood meal. During this journey, viruses encounter physical, cellular, microbial, and immunological barriers, which are influenced by the genetic background of the mosquito vector. This figure was created using BioRender.com.

“Vector competence” (VC) refers to the intrinsic ability of a vector to transmit a pathogen and comprises myriad complex processes and barriers within the vector itself. Scientific investigation into the inner drivers of VC has been a long-standing goal of the field, particularly towards exploiting vulnerabilities that interrupt virus transmission (Hardy et al., 1983). A series of excellent reviews have covered specific components of VC: the genetic basis for VC (Beerntsen et al., 2000), physical barriers (Hardy et al., 1983; Franz et al., 2015), mosquito immunity (Bartholomay and Michel, 2018; Kumar et al., 2018; Lee et al., 2019; Tikhe and Dimopoulos, 2021), microbiome (Cansado-Utrilla et al., 2021), and how VC contributes to the overall vectorial capacity of a vector for transmitting a pathogen (Kramer and Ciota, 2015). Over the past several decades, our understanding of how these barriers work together to result in a susceptible or refractory phenotype and drive virus evolution has become much more refined. Significant technological advancements in recent years have allowed researchers to further probe even more deeply into the mechanistic underpinnings of VC at the cellular and molecular level and begin synthesizing information on how one process or mechanism impacts or triggers another. The focus of this review is on key breakthroughs and advancements over the past decade, in our understanding of processes that intrinsically drive mosquito VC for arboviruses (**Figure 1**). Special attention is given to how these mechanisms are interlinked and influence virus evolution.

## 2 The genetic basis for vector competence

### 2.1 Foundational studies

A driving influence behind research into the genetic basis for VC has been germline transformation: the engineering of vectors to be refractory to virus transmission by driving resistance genes into wild populations (Collins and James, 1996; Terenius et al., 2008). Foundational to this line of research is knowledge of what genes govern VC, so this phenotype can be manipulated. Historically, loci associated with a susceptible or refractory phenotype have been identified through complex genetic crossing experiments that allowed researchers to identify marker loci that were linked to genes associated with pathogen susceptibility. During the 1990s, molecular genetic linkage maps were developed for several key arbovirus vectors using a variety of breakthrough techniques. These methods included cytogenetic analysis (e.g. polytene chromosomes), fluorescent *in situ* hybridization, and molecular methods (e.g. microsatellites, RFLP), and focused on *Aedes aegypti* (Munstermann and Craig, 1979; Severson et al., 1993; Antolin et al., 1996)*, Culex pipiens* (Mori et al., 1999), *Armigeres subalbatus* (Ferdig et al., 1998), and *Anopheles gambiae* (Holt et al., 2002). These pivotal advancements set the stage for the identification of quantitative trait loci (QTL) associated with the VC of these mosquito species for different disease agents (Zheng et al., 1993; Zheng et al., 1996). The sequencing of vector genomes and establishment of online repositories for housing and manipulating these genomic data followed suit (Amos et al., 2022; The VEuPathDB Project Team, 2023). Beerntsen et al. thoroughly reviewed the genetics of VC up through the year 2000, including investigations of immune gene expression in key tissues such as the midgut and salivary glands, however most of the literature to that point focused on the association of two primary model mosquitoes (*Ae. aegypti* and *An. gambiae*) with parasites (Beerntsen et al., 2000); research on the genetic basis for arbovirus transmission was still very much in its infancy.

Over the last 10 years, investigations of the genetic basis of VC for arboviruses have flourished. More so, key advancements of the past decade are attributable to the maturation of sequencing technology, mainstream availability of genomic and transcriptomic data, refined germline transformation approaches, and the discovery of endogenous non-retroviral genetic elements in mosquito genomes that influence VC. Additionally, significant advances have been made in dissecting the role of mosquito and virus genotype associations in determining VC. These topic areas are described in more detail below.

### 2.2 Technological advancements in genomics and transcriptomics promote breakthroughs in vector competence research

The ability to sequence genomes gave rise to the ability to generate transcriptomic data on the expression of messenger RNAs from different organisms. Technological advancements in the transcriptomics field evolved from looking at individual mRNA expression using expressed sequence tags (ESTs) or quantitative RT-PCR, to hundreds or thousands of transcripts using microarray hybridization, to full transcriptomes with RNA-Seq. These methods have been reviewed and described in detail (Lowe et al., 2017).

A key breakthrough in the application of transcriptomics technology to VC was the comprehensive publication of the *Ae. aegypti* transcriptome (Akbari et al., 2013; Matthews et al., 2018). High-resolution gene expression data were generated for this key vector species at dozens of time points throughout development in both somatic and germline tissues, and in association with biological sex and blood feeding. Key outcomes of this work also included the identification of endogenous small interfering RNAs and transposable elements. Having the ability to generate such high resolution transcriptomic data led to functional genomic studies, the design of gene drive strategies to integrate transgenes for pathogen refractoriness into wild mosquito populations, identification of sex- or tissue-specific gene expression patterns that would be relevant to manipulation of VC with transgenic approaches, and understanding the role of endogenous RNA elements in VC (Akbari et al., 2013). Moreover, transcriptomic profiling and comparison of different *Ae. aegypti* strains with natural differences in susceptibility to dengue virus (DENV) revealed a genetic basis in gene expression patterns associated with VC phenotype (Sim et al., 2013). Using whole genome microarrays, Sim et al. (2013) found that baseline immune gene expression was higher in *Ae. aegypti* mosquito populations that had a refractory phenotype as compared with mosquito strains that were susceptible to DENV. These results were confirmed with the knockdown of putative host factors followed by challenging mosquitoes with DENV. Transcriptomic studies of other key arbovirus vectors including *Ae. fluviatilis*, *Ae. vexans, Ae. albopictus*, and *Cx. pipiens* have followed with similarly impactful results (Caragata et al., 2017; Chung et al., 2020; Gamez et al., 2020; Martynova et al., 2022).

Transcriptomics of specific tissues and even individual cells relevant to VC have also yielded important insights and represent a burgeoning field. Ribeiro et al. generated a transcriptome for the salivary glands, also called a sialotranscriptome, of adult male and female *Cx. tarsalis* mosquitoes (Ribeiro et al., 2018). Advancements in sequencing technology allowed a more complete annotation of the genes expressed in the salivary glands, and genes putatively associated with blood feeding and nectar feeding were identified. Single-cell RNA-sequencing offers another technological advancement that will dissect the molecular responses to virus infection at the cellular level. Already, single-cell sequencing of hemocytes has revealed new insights into immune gene expression and hemocyte diversity in *An. gambiae* and *Ae. aegypti* mosquitoes (Raddi et al., 2020; Kwon et al., 2021). Differences in transcriptomic profiles have supported a genetic basis behind “susceptible” and “refractory” strains of *Ae. aegypti* mosquitoes. In the case of *Ae. aegypti*, differential gene expression was analyzed between refractory and susceptible mosquito strains challenged with DENV serotype 2 (Behura et al., 2014). The authors found transcriptional differences associated with vector genotype was explanatory of VC phenotype between mosquito strains. In particular, some key genes upregulated in DENV-susceptible strains were associated with endocytosis, peroxisome, autophagy and lysosome activity, protein and nucleotide binding, and metabolism. In contrast, genes upregulated in DENV-refractory mosquitoes were associated with energy metabolism, oxidative phosphorylation, mRNA surveillance, RNA transport, and innate immunity. The authors hypothesized that the DENV-responsive genes activated in the susceptible mosquitoes could be involved in viral entry and early cellular processes in the midgut epithelial cells, whereas in refractory mosquitoes gene activation was more associated with the energetic demands of fighting off the infection (Behura et al., 2014).

Transcriptomic technological advancements have also inspired innovative applications to field-based surveillance approaches. Metatranscriptomic analysis of individual mosquitoes has offered a tantalizing view into the complete prokaryote, viral, and eukaryote sequence profiles associated with individual insects. From such an analysis, one can characterize the virome of individual insects (Shi et al., 2020), confirm vector species identification, blood host source (if the mosquito was engorged), pathogen infection status, and microbial prevalence and co-infections (Chandler et al., 2015; Shi et al., 2019; Batson et al., 2021). Batson et al. performed metatranscriptomic profiling of 148 wild-caught mosquitoes from California; sequences were recovered from 24 known and 46 novel viral species; trypanosome, apicomplexan, and nematode parasites; *Wolbachia* endosymbionts, and vertebrate blood meals from wild and domestic animals (Batson et al., 2021). Relevant to VC, such data can be hypothesis-generating as far as identifying potential virus-vector associations or microbes that could either promote or interfere with arbovirus transmission. While geared towards biosurveillance applications, these valuable metatranscriptomic snapshots of individual mosquitoes in the field also provide data into the genetic background of the mosquito that would be permissive or refractory to harboring human-pathogenic organisms. This microbial ecosystem and genetic background of the mosquito also may place selective pressure on the diversity of infecting arboviruses, that could be characterized with these molecular approaches.

### 2.3 Endogenous non-retroviral elements

The technical advancements in genome sequencing have also revealed an abundance of transposable elements (TEs) and endogenous viral elements (EVEs) in mosquito genomes. In *Ae. albopictus*, TEs comprise 68% of the total genome (Chen et al., 2015). These mobile genetic elements represent sequences that have integrated into the genome of the host organism and are capable of independent movement and replication. Additionally, TEs also have a role in gene function and evolution, depending on the insertion site (Houé et al., 2019). EVEs are viral sequences that have integrated into the host genome as double stranded DNA (Katzourakis and Gifford, 2010). The origin of EVEs may or may not be from a retrovirus naturally capable of genomic integration. In mosquitoes, many of these EVEs appear to have been derived from flaviviruses, rhabdoviruses, and chuiviruses (Palatini et al., 2017; Whitfield et al., 2017), which have been able to integrate into the host genome through association with retrotransposons (Houé et al., 2019; Melo and Wallau, 2020). Houé et al. have thoroughly reviewed recent advances in understanding of TEs and EVEs in mosquitoes, of which much has been learned over the past couple decades (Houé et al., 2019). The linkage of EVEs with mosquito anti-viral immunity is discussed in section 4.2.

### 2.4 Mosquito genotype influences

The role of mosquito genetic background also influences VC. On a global scale, Vega-Rúa et al. used microsatellites to study the population structure of *Ae. albopictus* mosquitoes and correlate mosquito genetic background with VC for chikungunya virus (CHIKV) (Vega-Rúa et al., 2020). Six genetic lineages/clusters were characterized among the mosquito populations studied, and the VC of these different mosquito lineages trended towards adaptive coevolution for transmission of different lineages of CHIKV. Ultimately, the authors concluded that genotype-by-genotype interactions between mosquito and virus strains exert bidirectional selective pressure.

Even within the same isofemale line (family), mosquito genetic background may be influencing VC for different viruses in disparate ways. Novelo et al. investigated the impact of genetic variation on VC for DENV and CHIKV by employing a modified full-sibling design using viral RNA quantity as a proxy value for VC (Novelo et al., 2023). Mosquitoes from 37 families were reared separately; half of the siblings from each family were challenged with CHIKV and the other half DENV. Heritability was 40% for DENV, but only 18% for CHIKV, demonstrating that mosquito genetic background has a stronger influence on DENV viral RNA load and suggests that the genetic mechanism of viral control is variable by virus species. While these results are striking, a main limitation of this approach is that VC was estimated based on RNA quantity, and not the infection, dissemination, or transmission of live virus. RNA-Seq analysis identified two loci that varied consistently between mosquito families that exhibited high and low viral titers, including AAEL004181-PA, a distantly related member of the Salivary Gland Specific (SGS) genes. This gene has been purportedly involved in horizontal transfer from mosquitoes to *Wolbachia* endosymbionts (Korochkina et al., 2006; Woolfit et al., 2009), and this is the first report of its association with a VC phenotype. The influence of *Wolbachia* on VC is discussed in section 3.1.

## 3 Physical barriers

For a virus to be successfully transmitted, it must overcome the four physical (anatomical; physiological) barriers within a mosquito. These are: 1) midgut infection barrier, 2) midgut escape barrier, 3) salivary gland infection barrier and 4) salivary gland escape barrier (Hardy et al., 1983; Franz et al., 2015). Each of these barriers represents a significant bottleneck, stochastically reducing virus population size, diversity and complexity (Weaver et al., 2021). Bottleneck strength (the degree of which the virus population is reduced as it moves across the barrier) impacts the overall VC of a mosquito species for a given virus, contributing to whether the virus is ultimately transmitted in the saliva. While the presence of these physical barriers has long been recognized, current research continues to dissect and discover the mechanisms through which these barriers place selective pressure on viruses to influence both virus transmission and evolution.

### 3.1 Midgut

When evaluating midgut infection and escape barriers, mosquitoes are typically challenged with a single infectious blood meal, held for a set incubation period, and then assayed for viral dissemination. Inspired by the propensity of *Ae. aegypti* mosquitoes to take multiple blood meals during each gonotrophic cycle, Armstrong et al. challenged this traditional experimental design and offered *Ae. aegypti* mosquitoes a second, non-infectious blood meal following the initial infectious meal. In doing so, the extrinsic incubation period was shortened for viral dissemination from the midgut, and microperforations in the basal lamina resulting from midgut stretching were visually confirmed as a potential mechanism (Armstrong et al., 2020). Hence, the incorporation of ecologically significant parameters into the experimental design for VC assessments can be important for identifying underlying drivers of this process.

The molecular and cellular environment of the midgut epithelial cells also has a significant effect on the permissiveness to viral infection. Dong et al. identified differentially expressed genes in the midgut that were associated with viral dissemination (Dong et al., 2017). *Ae. aegypti* mosquitoes were fed either a saline or protein meal, with or without CHIKV. Trypsins, metalloproteinases, and serine-type endopeptidases were all expressed in the midgut following a blood meal, and potentially involved in midgut escape. The inclusion of CHIKV in either saline or protein blood meals only minorly affected the overall gene expression profile. The redox metabolic processes associated with ingestion of large amounts of heme and iron in a blood meal can also impact VC by presenting ingested viruses with the challenge of overcoming oxidative stress to establish infection. Often, reactive oxygen species (ROS) create a toxic cellular environment that inhibits viral infection, but cases exist in which ROS can promote viral infection (Paiva and Bozza, 2014). Oliviera et al. demonstrated that production of antioxidant catalase in the midguts of *Ae. aegypti* peaked at 24h post blood ingestion. The protection to midgut cells offered by catalase also facilitated midgut infection with DENV-4, but not Zika virus (ZIKV) (Oliveira et al., 2017). Similarly, Liu et al. found that the presence of ROS was significantly detrimental to the establishment of DENV-2 infection in *Ae. aegypti* mosquitoes, and that susceptibility to virus infection was increased when ROS were inhibited with vitamin C (Liu et al., 2016). Antiviral immune pathways triggered in the midgut epithelial cells also play a significant role in midgut infection and escape and are discussed below.

### 3.2 Salivary glands

For virus transmission to occur, virus particles must not only enter the salivary glands from the hemocoel, but also egress through expectoration in the saliva. These processes are not necessarily coupled, in that salivary glands may be permissive to virus infection, but not to escape. Stauft et al. reported that *Cx. tarsalis* mosquitoes infected with either the McMillan or IMP181 strains of Western equine encephalitis virus (WEEV) were equally permissive to infection of the salivary glands by both virus strains. However, IMP181 was more readily transmitted (Stauft et al., 2022). Upon follow-up infection using recombinant WEEV strains, salivary gland escape of the McMillan strain was partially rescued by inclusion of particular components of the IMP181 structural polyprotein (Stauft et al., 2022). Just like the midgut, entry and escape of virus particles from the salivary gland tissue are unique processes that present different selective pressures on viruses.

To investigate the role of mosquito genetics at the salivary gland barrier, a half-sib quantitative genetic analysis of an *Ae. aegypti* strain from Mexico assessed the genetic basis for a salivary gland infection and escape barriers (Sanchez-Vargas et al., 2021). Consistent with the findings of Novelo et al., the heritability of VC at the level of the salivary glands was virus species-dependent. Specifically, a salivary gland infection barrier was evident when mosquitoes were infected with DENV but not ZIKV or CHIKV. In contrast, there was a moderate salivary gland escape barrier when mosquitoes were infected with ZIKV or CHIKV but not DENV. The influence of genetic factors was also more pronounced when viral titers were low (Novelo et al., 2023). Taken together, mounting evidence supports an underlying genetic basis for VC, but that is variable across mosquito and virus species. These virus-vector genotype associations account for a significant proportion of the underlying transmission potential.

More research is needed to elucidate the mechanisms of viral entry and egress from the salivary glands. To this end, some important tools have recently been developed to facilitate study of the salivary gland barriers. The salivary gland proteome for *Ae. aegypti* was published in 2017, representing an important step forward in studying virus-vector interactions. This dataset comprises 128 proteins, 29 of which have purported immune functionality, and 15 are secretory in nature (Dhawan et al., 2017). Protocols have also been developed for performing immunohistochemistry of the mosquito salivary glands, which will provide important visualization of viral and vector proteins (Martin-Martin et al., 2022). With specific proteins in mind, one can interrogate the specific lobes and cells of the salivary glands important to viral infection, as well as which mosquito proteins are critical to these interactions.

## 4 Microbial interactions

A mosquito’s microbiome (including bacterial, viral, and fungal agents) can greatly impact its VC for arboviruses. The microbiome is not fixed, but comprises a community within the vector that is dynamic and in a state of continual flux due to the influence of extrinsic and intrinsic factors such as food availability and environmental conditions (Novakova et al., 2017; Bogale et al., 2020; Medeiros et al., 2022; Accoti et al., 2023). The effect of the microbiome on mosquito VC for arboviruses challenges the traditional definition of VC as the interaction of “intrinsic” genetically encoded fixed traits of the vector (e.g. mosquito proteins, genetic material, and other biomolecules on and in cells and tissues) with virions, virus genetic material or other virus-encoded proteins. The microbiome affects VC through direct interaction with arboviruses or by indirect influence on virus replication or dissemination via resource competition. A mosquito’s microbiome could also directly or indirectly influence the production of mosquito DNA, RNA, proteins, and other biomolecules, such as lipids, that an arbovirus relies on. These macromolecules, such as innate immune factors, could target the arbovirus or its genetic material. Importantly, alterations in biomolecules may be due to sub-lethal effects that pathogenic microbes induce in the mosquito. This review will focus on both artificial transinfections as well as natural commensal and pathogenic bacterial species, insect-specific viruses (ISVs), and fungi that affect VC. It is also noteworthy that some groups are working to genetically manipulate these microbes, for example, to express peptides that make the microbes more lethal to the mosquito or to block pathogen transmission in the mosquito (Fang et al., 2011; Reveillaud et al., 2019).

### 4.1 Wolbachia

A focus of much vector microbiome research is the bacterial genus *Wolbachia*, which is a naturally occurring intracellular bacterium in the reproductive tissues of many arthropods including some mosquito species. *Wolbachia* has adapted mechanisms of manipulating arthropod reproduction to achieve increased inheritance rates in a population, reviewed by Werren and colleagues (Werren et al., 2008). In mosquitoes, the presence of *Wolbachia* has varying effects on VC. The phenomenon of cytoplasmic incompatibility causes sperm from *Wolbachia*-infected males to be incompatible with *Wolbachia-*uninfected females, preventing reproduction completely. Cytoplasmic incompatibility drives *Wolbachia* into a mosquito population, making it an attractive target for reducing the VC of a population.

The effect of *Wolbachia* on VC differs between viruses and vector species. Experimental work has demonstrated *Wolbachia*-mediated pathogen blocking impacts *Ae. aegypti* VC for DENV, CHIKV, ZIKV, and Mayaro virus (MAYV) replication, dissemination and transmission (Moreira et al., 2009; Bian et al., 2010; Ferguson et al., 2015; Dutra et al., 2016; Pereira et al., 2018; Chouin-Carneiro et al., 2020; Guo et al., 2022). The mechanism of *Wolbachia-*mediated pathogen blocking in *Aedes* mosquitoes is not yet fully understood. Some studies demonstrate upregulation of innate immune pathways in *Wolbachia*-infected *Aedes* mosquitoes (Bian et al., 2010; Pan et al., 2012), while another found that DENV inhibition by *Wolbachia* was independent of Toll pathway activity (Rancès et al., 2013). Alternatively, *Wolbachia* infected *Cx. tarsalis* mosquitoes were more susceptible to West Nile virus infection than wild-type controls. The *Wolbachia*-infected mosquitoes were also found to have suppressed Toll pathway activity, presenting a potential causative mechanism (Dodson et al., 2014). *Wolbachia* was found to have minimal impact on Rift Valley fever virus infection in *Cx. tarsalis* (Dodson et al., 2017). More recently, experimental infections have demonstrated mixed effects of *Wolbachia* on alphavirus infection in *Ae. aegypti* including enhancement of Sindbis virus, no impact on O’nyong-nyong virus, and inhibition of MAYV in a *Wolbachia* strain-dependent manner (Dodson et al., 2023).

The natural effects of *Wolbachia* infection on arboviruses can be harnessed as a field application to reduce arbovirus transmission in endemic regions; this concept has been tested in field-application studies repeatedly in the last decade. There are two targetable outcomes for field-application of *Wolbachia* deployment: population replacement (supplantation of the wild-type mosquito population in a region with *Wolbachia*-infected mosquitoes to achieve reduced VC for endemic pathogens) and population suppression (massive release of male *Wolbachia*-infected mosquitoes to interrupt reproduction and reduce or eliminate the target mosquito population). The propensity of *Wolbachia* to drive population replacement has been demonstrated in multiple field studies. Notably, a field release of infected *Ae. aegypti* mosquitoes in Australia resulted in stable natural population infection for 10 years as of 2022 with reduced DENV transmission in the treatment areas (Hoffmann et al., 2011; Hoffmann et al., 2014; Ryan et al., 2019; Ross et al., 2022). Similar field trials have seen successful *Ae. aegypti* population replacement in other parts of Australia, Brazil, Colombia, Malaysia, Indonesia, and Vietnam, with some reporting a reduction in endemic arbovirus transmission (CHIKV and DENV in Brazil, DENV in Australia, Indonesia, and Malaysia) (O’Neill et al., 2018; Nazni et al., 2019; Velez et al., 2019; Tantowijoyo et al., 2020; Pinto et al., 2021; Utarini et al., 2021). A current criticism against population replacement, is that it may prevent future deployment of population suppression in the same area.

*Wolbachia* naturally occurs in many mosquito species including vector species of diverse arboviruses; *Wolbachia* surveillance in wild mosquito populations has been reviewed (Inácio da Silva et al., 2021). Considering the variability in lab-produced results regarding the impact of *Wolbachia* on VC, it is difficult to speculate how natural infection may be impacting the VC of these populations. For instance, a microbiome study of wild *Ae. aegypti, Ae. albopictus*, and *Cx. quinquefasciatus* mosquitoes in Thailand, where DENV, CHIKV, ZIKV, and Japanese encephalitis virus are endemic, found them to be infected with *Wolbachia* (Thongsripong et al., 2017). Would transmission of these viruses be higher in the area if the mosquito populations were not infected with *Wolbachia*? The field applications studies of population replacement suggest that there might be.

Field deployments of the population suppression method have also been successful, including an *Ae. albopictus* effort in China and suppression of *Ae. aegypti* in Singapore and the USA (Florida and California) (Mains et al., 2019; Zheng et al., 2019; Crawford et al., 2020; Consortium and Ching, 2021). The use of *Wolbachia* as an arboviral transmission control mechanism in the field illustrates how factors intrinsic to the mosquito such as the microbiome can be hijacked to reduce VC in a large-scale way.

### 4.2 Other bacterial endosymbionts

The effect of other microorganisms has also been characterized in mosquitoes and studied regarding their effect on VC. The species of bacteria that make up the microbiome of mosquitoes vary greatly between and within mosquito species. Dominant bacterial genera colonizing *Ae. aegypti* include *Leptothrix, Methylobacterium, Enterobacter, Methylotenera*, *Escherichia, Shigella*, and *Sphingomonas* (Lin et al., 2021). However, for *Ae. albopictus*, the gut microbiome is concentrated into fewer genera, with bacteria such as *Wolbachia, Bacillus, Methylobacterium* and *Enterobacter* being dominant above other genera (Lin et al., 2021). Given how different bacterial species interact with arboviruses and the mosquito’s immune system, these differing microbiomes may play a significant role in VC. A 2012 study showed that various species of bacteria isolated from the midguts of field mosquitoes can have varying effects on DENV titers, depending on the bacterium’s ability to colonize the midgut and affect the mosquito immune system (Ramirez et al., 2012). *Proteus* sp. (Prsp-P) effectively colonized the midgut and increased transcript abundance of many antimicrobial genes including cecropin, gambicin and attacin, leading to a subsequent decrease in DENV titers. Similarly, bacteria in the genus *Chromobacterium* (Csp_P) also decreased DENV titers when fed to *Ae. aegypti* mosquitoes (Ramirez et al., 2012). Further work by the same group revealed that the bacteria co-express a protease and an aminopeptidase that functioned in concert to degrade the envelope protein of DENV. This activity likely interferes with viral attachment to cells (Saraiva et al., 2018).

### 4.3 Insect-specific viruses

The mosquito’s virome is another vital component of the microbiome. Insect-specific viruses (ISVs) have been widely characterized in mosquitoes, beginning with cell-fusing agent virus (CFAV) in *Ae. aegypti* (Stollar and Thomas, 1975). In a 2017 study, CFAV was shown to increase replication of DENV in mosquito cell lines (Zhang et al., 2017). However, in 2019, a contrasting study showed that CFAV reduced dissemination rates for DENV and ZIKV in *Ae. aegypti* (Baidaliuk et al., 2019). Other widespread ISVs, like Humaita-Tubiacanga virus (HTV) and Phasi Charoen-like virus (PCLV) increased competence for DENV in *Ae. aegypti* by blocking downregulation of the proviral host factor histone H4 (Olmo et al., 2023). The impact of other insect-specific viruses on VC, such as Culex flavivirus (CxFV), are still being debated given conflicting results regarding the relationship between WNV and CxFV (Bolling et al., 2011; Newman et al., 2011; Crockett et al., 2012). Further research on ISVs is essential to fully characterize their possible effects on arboviral competence.

### 4.4 Fungi

There are many fungi that contribute to the microbiome of mosquitoes. This includes yeast of the genera *Pichia* and *Canadia*, which were found in both *Aedes* and *Culex* mosquitoes (Gusmão et al., 2010). Although there has been limited work demonstrating the effect of these organisms on VC, in a 2017 study, researchers identified the naturally occurring fungus *Talaromyces* in the midguts of *Aedes* mosquitoes. Furthermore, they demonstrated that the presence of the fungi is correlated with an increase in DENV titer in the midgut (Angleró-Rodríguez et al., 2017). Further research is needed to characterize the effects of other fungi on arboviral competence. There is also potential to use fungi as a transgenic vector to affect pathogens in the mosquito, as shown relating to *An. gambiae* and malarial parasites (Fang et al., 2011).

## 5 Cellular immunity and antiviral responses

Mosquito antiviral immunity is mediated by cellular and molecular mechanisms. For a recent in-depth overview of mosquito antiviral immunity, please see the 2021 review by (Tikhe and Dimopoulos, 2021). Briefly, the cellular arm of the mosquito immune response consists of hemocytes, of which there are three subtypes: prohemocytes, granulocytes, and oenocytoids. Granulocytes comprise the majority of the hemocyte population (80-95%) and are a primarily phagocytic cell type, but they also contribute to opsonization, prophenyloxidase (PPO) production, and antimicrobial peptide production. The function of prohemocytes is largely undefined, though the two hypotheses are 1) prohemocytes are a progenitor cell type (Castillo et al., 2006) or 2) prohemocytes result from the asymmetric mitotic division of granulocytes (King and Hillyer, 2013). Oenocytoids are the primary producers of PPO, which is essential for the process of melaninization, a defensive response that encases invading pathogens to prevent dissemination. The primary intracellular signaling pathways that participate in antiviral immunity are the JAK-STAT pathway, the IMD pathway, and the Toll pathway. These pathways are activated upon recognition of pathogen-associated molecular patterns, and a signaling cascade follows to induce the transcription of antimicrobial genes. These pathways have demonstrated abilities to impact viral replication, but it is unclear and somewhat difficult to determine how they have shaped the evolution of arboviruses in the face of host alternation (Tikhe and Dimopoulos, 2021).

The RNA interference (RNAi) pathway is activated by the presence of double-stranded RNA, a replicative intermediate of RNA viruses, and results in sequence-specific degradation of RNA. There are three branches of RNAi that differ primarily by the method of acquisition of the template RNA. In the small interfering RNA (siRNA) pathway, the cytoplasmic dicer protein detects and digests double-stranded RNA (dsRNA) into small fragments which are then used as templates by RISC (RNA-induced silencing complex) to recognize and destroy RNA with the same sequence. The piwi-interacting RNA (piRNA) pathway obtains guide RNAs from genetic loci containing clusters of partial sequences from viruses and transposons. The piRNA and siRNA pathways have been demonstrated to have a direct role in mosquito antiviral immunity. The primary function of the micro-RNA (miRNA) pathway is gene regulation, with guide RNAs being produced from genetic loci in the nucleus of the host cell. A direct anti-viral role for the miRNA pathway has not been demonstrated, though genomic analysis of the *Ae. aegypti* genome has identified putative anti-viral miRNAs (Yen et al., 2019). Alterations in miRNA profiles during viral infection indicate that the pathway may be involved in the host cell response and regulation of innate immune pathways (Campbell et al., 2014; Avila-Bonilla et al., 2017; Saldaña et al., 2017; Su et al., 2017). miRNA may, therefore, impact the host cell permissiveness and hence VC of the mosquito, but this has yet to be demonstrated conclusively.

### 5.1 The role of mosquito hemocytes during viral infection remains a knowledge gap in understanding mosquito anti-viral immunity

The role of mosquito hemocytes in antiviral immunity is poorly characterized; more is known about their role during infection by protozoans, bacteria, and fungi. Even the overt role of hemocytes in viral infection is up for debate: do they contribute to viral dissemination in the mosquito? Hemocytes are the only cell type with the ability to move throughout the entire vector, making them an attractive target for viral infection to promote dissemination; a number of arboviruses have demonstrated tropism for mosquito hemocytes (Salazar et al., 2007; Parikh et al., 2009; Cheng et al., 2016). While direct evidence for the role of mosquito hemocytes in controlling viral infections is lacking, they have several functions that could potentially contribute to antiviral immunity including initiation of melaninization, production of antimicrobial peptides, pathogen-associated molecular pattern (PAMP) recognition, and apoptosis induction. Work in *Drosophila* has illuminated a role for hemocytes as essential mediators of the RNAi response (Tassetto et al., 2017). While demonstration of dsRNA uptake by mosquito hemocytes suggests they may also play such a role, evidence of downstream RNAi activity is lacking (Airs et al., 2023). A number of sequencing experiments have also identified interconnectivity between the molecular innate immune pathways (IMD, Toll, JAK-STAT) and hemocyte activity during *Plasmodium* and bacterial infection of *Anopheles spp*, but this has not been replicated in a mosquito-virus system (Ramirez et al., 2014; Yan et al., 2022).

The development and increasing availability of single-cell sequencing has improved the granularity of understanding of mosquito hemocyte populations. Raddi et al. used single-cell sequencing to examine the transcriptome of hemocytes from *Ae. aegypti* and *An. gambiae*, two important vectors (Raddi et al., 2020). From the resulting transcriptome data, the group was able to characterize mosquito hemocytes based on transcriptional markers and provide insights into the hemocyte population lineage and structure, including the identification of a new granulocyte subtype in *An. gambiae* but not in *Ae. aegypti*, the megacyte. Further experiments characterized how the hemocyte population in *An. gambiae* was altered following infection with human malaria pathogens. They describe increased hemocyte circulation, granulocyte activation and proliferation, and prohemocyte differentiation in response to infection with *Plasmodium*. A few other studies have used single-cell sequencing in combination with functional assays to characterize hemocyte populations from *An. gambiae* (Severo et al., 2018; Kwon et al., 2021).

Limited previous work has suggested a role for mosquito hemocytes during viral infection (Leite et al., 2021). Even with few available publications, already single-cell sequencing has revealed differences in hemocyte population structure between important vector species that could play a role in differing VC. With the majority of this research targeting anti-*Plasmodium* immunity in *An. gambiae*, there is much to be learned about how hemocytes influence and are influenced by viral infection in other important vector species through the use of single-cell sequencing.

### 5.2 The piRNA pathway and endogenous viral elements

In recent years, key research advancements in mosquito immunology have described the role of EVEs in modulation of VC, and the mechanisms through which this happens. The genomic integration of EVEs and subsequent influence on antiviral immunity is intimately associated with the piRNA pathway, which in turn influences VC. The piRNA pathway is primarily recognized for its role in anti-transposon activity, and the role of piRNAs in guiding PIWI proteins in cleaving target RNA sequences (Ozata et al., 2019). However, this pathway has evolved anti-viral defense properties in mosquitoes and has a notable association with EVEs (Palatini et al., 2017; Whitfield et al., 2017). EVEs have been discovered in *Ae. aegypti* mosquitoes from geographically disparate populations around the world, and multiple groups have now reported the genomic association of EVEs with piRNA clusters (Tassetto et al., 2019; Aguiar et al., 2020; Crava et al., 2021). Moreover, it was EVE-derived piRNAs that associated with PIWI4 to restrict acute and persistent secondary arbovirus infections in mosquito cells rather than piRNAs derived from actively replicating virus (Tassetto et al., 2019). In this way, EVEs represent a genetic element that plays a very directed role in controlling viral replication of the cognate, secondarily infecting virus from which the EVE was derived. Suzuki et al. demonstrated this functional association between EVEs and piRNA-driven antiviral immunity by knocking out a specific EVE in *Ae. aegypti* derived from cell fusing agent virus (CFAV) using CRISPR-Cas9 (Suzuki et al., 2020). Replication of CFAV in mosquito ovaries was significantly increased in the EVE-knockout mosquito line (Suzuki et al., 2020). So, what is the advantage to the mosquito or virus, for this seemingly self-sacrificial mechanism for a virus to inhibit replication of itself? Goic et al. provided experimental evidence that EVEs are important to promoting mosquito survival against arboviral infection and allowing tolerance of persistent infection. Mosquitoes treated with the reverse transcriptase inhibitor azidothymidine (AZT) succumbed to infection with CHIKV, whereas untreated mosquitoes survived (Goic et al., 2016). Hence, the control of viral infection provided by EVEs may ensure both the survivorship of the mosquito as well as the transmissibility of the virus.

### 5.3 The arms race between arboviruses and RNAi has created a balance that promotes viral persistence and mosquito survivorship

RNAi has been demonstrated as essential to controlling arbovirus pathogenicity in the invertebrate host. *Ae. aegypti* mosquitos with a detrimental mutation in the essential RNAi gene *Dcr-2* gene developed a disease phenotype upon infection with Sindbis virus, DENV-1, DENV-4, and YFV (Samuel et al., 2023). Merkling et al. corroborated these similar experiments and found a disease phenotype presented in *Dcr-2* knockout *Ae.* aegypti infected with CHIKV (Merkling et al., 2023). Interestingly, this work also indicated that knockout of *Dcr-2* was not associated with a change in overall vector competence. Alternatively, over-expression of RNAi genes *Dcr-2* and *R2d2* resulted in a significantly reduced replication of DENV, ZIKV, and CHIKV (Dong et al., 2022). This illustrates that a delicate balance has been struck between mosquito immunity and viral control of mosquito immunity that allows both the virus and vector to survive and continue the viral life cycle.

Several arboviruses have been demonstrated to generate viral suppressors of RNAi (VSRs) that function in mosquito models, including CHIKV non-structural proteins nsP2 (nsP2) and nsP3 (nsP3) which have RNAi suppression activity in both insect and mammalian cells. RNA binding motifs in these proteins are highly conserved across 15 species of alphavirus, indicating likely conserved VSR activity (Mathur et al., 2016). Qiu et al. produced evidence that flavivirus protein NS2A is a conserved NSR as well. DENV-2 NS2A was demonstrated to sequester dsRNA from the dicer endonuclease in both mammalian and insect cells (Qiu, 1992). The same study confirmed RNAi suppression by the NS2A protein of DENV-1, -3, and -4, and ZIKV, JEV, and WNV in human cells. JEV NS2A was additionally screened for and found to have NSR activity in mosquito cells. In contrast, Schnettler et al. found no NSR activity when they screened WNV non-structural proteins, though they only examined NS2A as part of the replication complex rather than individually (Schnettler et al., 2012). This group investigated the RNAi suppressive activity of the sfRNA of DENV, Kunjin virus, and WNV. Their results showing increasing concentration of WNV sfRNA is correlated with reduced dicer cleavage of dsRNA suggests that the sfRNA may be acting as a competitive substrate for dicer. The same activity was subsequently demonstrated for DENV-1 sfRNA as well. By developing means of controlling the RNAi pathway, viruses dampen the mosquito’s immune response and create an environment that allows the virus to survive without negative impacts on the vector that would reduce VC.

## 6 Vector competence and viral evolution

The processes driving VC play an important role in the evolution of arboviruses. There are multiple facets to the relationship between mosquitoes and viruses that drive viral evolution, including selective pressures, stochastic bottlenecks, co-evolution, and host range. These factors can influence arboviruses via positive, purifying, or diversifying selection. Positive and purifying selection represent opposite effects; positive selection fixes beneficial mutations in a population whereas purifying selection removes deleterious mutations. Diversifying selection occurs when phenotypes differing from the majority phenotype are favored, resulting in higher population diversity. Arboviruses are RNA viruses, which have high mutation rates compared to DNA viruses, due to their lack of proof-reading enzymes. The RNA-dependent RNA polymerase (RdRp), the enzyme responsible for replicating viral RNA, has an error rate of approximately 10^-3^ to 10^-6^ mutations for nucleotide copied, resulting in approximately one mutation per viral genome (Selisko et al., 2018; Caldwell et al., 2022). This results in a mutant swarm – a highly diverse and dynamic collection of closely related, but genetically distinct viral variants. This viral genetic diversity contributes to the adaptability of flaviviruses, especially as they alternative between their vertebrate and invertebrate hosts, and can be important in evading host immune responses, adapting to new hosts and species, and overall population stability (Weaver et al., 2021).

### 6.1 Stochastic bottlenecks

It has been shown for many virus-mosquito pairings (e.g., VEEV in *Cx. taeniopus*, ZIKV in *Ae. aegypti*, WNV in *Cx. tarsalis*, *Cx. quinquefasciatus* and *Ae. aegypti*), escape of the virus from the midgut into the haemocoel imposes a stringent bottleneck, significantly reducing virus population diversity and complexity (Forrester et al., 2012; Weger-Lucarelli et al., 2018; Fitzmeyer et al., 2023). Additional studies have quantified the reduction of viral complexity as the virus moves across the other mosquito barriers, demonstrating significant bottlenecks at each step of systemic mosquito infection (Smith et al., 2008; Ciota et al., 2012). Because this process is stochastic, these bottlenecks can undo any natural selection (positive or purifying) that has shaped virus populations within the mosquito tissues by randomly selecting variants that overcome the barrier (Weaver et al., 2021). Importantly, recent studies with WNV have demonstrated that while VC does not impact the overall diversity of transmissible WNV populations, vectors with higher competence are more likely to transmit rare variants (Fitzmeyer et al., 2023).

### 6.2 Natural selection

Arboviruses are under a uniquely broad set of selective pressures given that they must be capable of escaping or antagonizing both the innate and adaptive immune responses of two distantly related hosts. Thus, any adaptations acquired to counter one host’s immune response must either improve or have no effect on the ability of the virus to escape the immune response of the other host to maintain fitness. As the evolution of arboviruses is inextricably linked to the selective pressures of two different hosts, it is challenging to determine whether adaptations leading to increased fitness arose due to the selective pressure of the vertebrate or invertebrate host immunity. Furthering our understanding of the interplay between viruses and the invertebrate immune response is necessary to dissect the mechanism by which arboviruses evolve and emerge.

Interactions between a virus and vector create selective pressures that shape virus populations, with higher fitness variants (e.g., those that replicate more efficiently within the mosquito), becoming more dominant within the virus mutant swarm. Both positive selection (e.g., beneficial viral variants becoming more prevalent in the population) and purifying selection (e.g., deleterious or harmful viral variants being removed from the population) play significant roles in shaping arbovirus populations within mosquitoes (Ciota and Kramer, 2010). Unlike bottlenecks, these selective events are not stochastic.

Extensive work has characterized selective pressures exerted on arboviruses in their mosquito vectors, and across many virus and mosquito species, it has been shown that virus populations can evolve dramatically during a single infection (Jerzak et al., 2007; Brackney et al., 2009; Sim et al., 2015; Grubaugh et al., 2016; Lequime et al., 2016). Mosquito immunity (discussed above) has been shown to result in selective pressures on virus populations within mosquitoes (Prasad et al., 2013). For example, in WNV-infected *Culex* mosquitoes, rare viral haplotypes have an advantage against unmutated viral genomes, due to their poor match against guide strands and targeting by the RNA-induced silencing complex (RISC). This allows rare viral variants to replicate, leading to diversified populations (Brackney et al., 2009; Brackney et al., 2015). Mosquito host genetics and genotypes (discussed above), which can strongly impact VC, has been shown to alter rates of natural selection and genetic diversity in DENV infected *Ae. aegypti* (Lambrechts and Lequime, 2016; Lequime et al., 2016). There is also evidence of positive selection, purifying selection and other types of virus population evolution has been shown in SLEV-infected *Cx. pipiens* (Ciota et al., 2009), VEEV-infected *Ae. aegypti* (Coffey et al., 2008), ZIKV-infected *Ae. aegypti* (Riemersma et al., 2021). Any changes to virus populations can result in the emergence of new strains or variants, which can have altered properties, including differences in virulence, transmissibility by mosquitoes, or the ability to infect different hosts or species.

### 6.3 Virus and mosquito coevolution

There is a dynamic interplay between the coevolution of mosquitoes and the viruses they transmit, which is characterized by reciprocal adaptations over evolutionary time. Mosquitoes have evolved complex anti-viral immune mechanisms to limit and combat viral infection (Cheng et al., 2016). In response, arboviruses have developed robust strategies to evade and combat mosquito immune defenses (Blair and Olson, 2014; Sim et al., 2014). This coevolution over millions of years, has shaped both virus arbovirus and mosquito genetic diversity. As arboviruses systemically and persistently infect a mosquito host, they can adapt to optimize their ability to infect and replicate within mosquito tissues and improve their ability of being successfully transmitted in the saliva (Ciota and Kramer, 2010; Novella et al., 2011; Lambrechts, 2023). There are many examples of host adaptation shaping virus populations and evolution. For example, when WNV was passaged in *Cx. pipiens* mosquitoes, it was found that the passaged virus resulted in higher infection, dissemination and transmission rates, and there were multiple amino acid coding changes in the viral genome likely causing the enhanced VC (Ciota et al., 2008). Similarly, JEV genotype I displaced genotype III as the dominant genotype, likely due to coevolution to the mosquito vector, resulting in increased mosquito infection, and a shorter extrinsic incubation period (Schuh et al., 2014).

### 6.4 Host range expansion and emergence of new viral variants

When viruses evolve to be highly competent in a particular mosquito species, it can become restricted to the geographic range of that mosquito. However, climate change, alterations in land use, and increase human mobility are all contributing to the expanding geographic range of many mosquito species. This leads to an increased geographic range of the viruses these mosquitoes transmit and can result in arbovirus interactions with new mosquito species.

As arboviruses encounter novel mosquito vectors, they can rapidly adapt to the novel species, leading to new variants with epidemic potential. For example, CHIKV is primarily transmitted by *Ae. aegypti* species of mosquitoes. Asian CHIKV strains were constrained to these mosquitoes, however an African lineage CHIKV contained an amino acid change allowing it to efficiently infect and be more quickly transmitted by *Ae. albopictus* (Tsetsarkin et al., 2007; Dubrulle et al., 2009; Tsetsarkin et al., 2011). This allowed African lineage CHIKV to occupy the *Ae. albopictus* vector niche, resulting in major epidemics of severe disease. Similarly, rapid CHIKV microevolution occurred during an Indian Ocean outbreak, likely because of successful adaption to local *Ae. albopictus* (Schuffenecker et al., 2006). Similarly, as WNV was introduced to the United States, and subsequently spread across the country, a newly emergent genotype was found to be transmitted sooner and more efficiently by the local *Culex* species mosquitoes (Moudy et al., 2007).

### 6.5 RNAi influences viral evolution and emergence through diversifying selection

The RNAi pathway has been explored as a driver of diversifying selection of arboviruses as they replicate in the mosquito host. RNAi has a negative frequency-dependent selection effect, as the process of the siRNA pathway will destroy RNA that is identical to the loaded guide strand while leaving RNA containing mutations in the reference region intact. Based on probability, the RNA of genetic variants constituting larger proportions of the viral population will more frequently be degraded and used as guide RNA in the siRNA pathway, thus selecting for less frequent variants. A study saw significantly reduced viral diversification following the passage of WNV in RNAi-depleted *Drosophila* cells compared to RNAi-competent and RNAi-stimulated *Drosophila* cells (Brackney et al., 2015). Another experiment demonstrated that the viral population represented in mosquito salivary glands after it has travelled through the mosquito and experienced various stochastic selections via physical barrier bottlenecks and RNAi diversifying selection is characterized by greater genetic diversity than the population found in the avian host (Grubaugh et al., 2016). In the case of WNV, this increased population diversity was demonstrated to reduce viral fitness as the virus then re-enters the avian host, in which strong purifying selection occurs (Jerzak et al., 2005). While much of the genetic variance in the viral output from the mosquito is lost during subsequent replication in an avian host, this diversification allows arboviruses to explore a large adaptive landscape during each host alternation cycle.

## 7 Conclusions

The intrinsic factors underlying virus infection, dissemination, and transmission by mosquitoes are complex and interconnected. Technological advances in molecular biology, virology, and immunology have significantly refined our understanding of what mechanisms influence VC within the mosquito down to the tissue, and even cellular level. Importantly, though discussed separately above, these various drivers of VC are functionally intertwined. In the midgut, infecting viruses must interact with endosymbiotic bacteria, viruses, and fungi and overcome oxidative stress upon ingestion with a blood meal. These microbial interactions as well as the presence of double-stranded RNA from replicating viruses with the midgut epithelium all trigger the mosquito immune system in various ways that influence VC. Immune gene expression is also active in the salivary glands, which provide unique barriers to viral entry and egress. Whether or not the mosquito outwardly displays a susceptible or refractory phenotype is further dependent on a heritable genetic background, and transcriptional differences of immune genes across tissue types or mosquito strains. Endogenous viral elements integrated into the genome function to temper viral infection through the piRNA immune pathway. Through all of these interactions occurring within the mosquito, stochastic bottlenecks and selective pressures on the virus at each tissue barrier drive virus-vector co-evolution and result in the transmission outcomes we observe in the field.

## Author contributions

JL: Writing – original draft, Writing – review & editing. EG: Conceptualization, Writing – original draft, Writing – review & editing. JR: Conceptualization, Writing – original draft. AG: Conceptualization, Writing – review & editing. BF: Supervision, Writing – review & editing. GE: Supervision, Writing – review & editing. RK: Conceptualization, Visualization, Writing – original draft, Writing – review & editing.
