# Corrigendum: Intrinsic factors driving mosquito vector competence and viral evolution: a review

**DOI:** 10.3389/fcimb.2024.1375638

**Published:** 2024-02-07

**Authors:** Juliette Lewis, Emily N. Gallichotte, Jenna Randall, Arielle Glass, Brian D. Foy, Gregory D. Ebel, Rebekah C. Kading

**Affiliations:** ^1^ Center for Vector-borne Infectious Diseases, Department of Microbiology, Immunology, and Pathology, Colorado State University, Fort Collins, CO, United States; ^2^ Department of Cellular and Molecular Biology, Colorado State University, Fort Collins, CO, United States

**Keywords:** transmission, barriers, arbovirus, antiviral immunity, microbiome, genomics, applications

In the published article, there was an error in the Funding statement. Part of the funding statement that included an NSF grant number was left out of the published article. The correct Funding statement appears below.

**FUNDING**

The author(s) declare financial support was received for the research, authorship, and/or publication of this article. RCK, and JL are receiving salary support from the United States Department of Agriculture, Agriculture Research Service (Project # 58-3022-1-001). EG was supported by funding to Verena (viralemergence.org) from the U.S. National Science Foundation, NSF BII 2213854. The content of the information does not necessarily reflect the position or the policy of the federal government.

The authors apologize for this error and state that this does not change the scientific conclusions of the article in any way. The original article has been updated.

